# Characterizing the Preferred Retinal Locus and Fixation Stability in Diabetic Macular Ischemia: A One-Year Study

**DOI:** 10.3390/vision9010020

**Published:** 2025-03-05

**Authors:** Alicia Lim, Wei-Shan Tsai, Sridevi Thottarath, Sarega Gurudas, Taffeta Ching Ning Yamaguchi, Elizabeth Pearce, Sobha Sivaprasad

**Affiliations:** 1 Barts and the London School of Medicine and Dentistry, London E1 2AD, UK; a.y.lim@smd20.qmul.ac.uk; 2 Moorfields Clinical Research Facility, NIHR Biomedical Research Centre, Moorfields Eye Hospital NHS Foundation Trust, London EC1V 2PD, UK; wei-shan.tsai@nhs.net (W.-S.T.); s.thottarath@nhs.net (S.T.); sarega.g@gmail.com (S.G.); 3 Hampshire Hospitals NHS Foundation Trust, Hampshire RG24 9NA, UK; 4 Boehringer Ingelheim, Binger Strasse 173, 55216 Ingelheim am Rhein, Germany; taffeta.yamaguchi@boehringer-ingelheim.com; 5 Institute of Ophthalmology, University College London, London EC1V 9EL, UK; lizzzpearce@gmail.com

**Keywords:** bivariate contour ellipse area, diabetic macular ischemia, diabetic retinopathy, fixation stability, microperimetry, optical coherence tomography, optical coherence tomography angiography, preferred retinal locus, retinal sensitivity, visual acuity

## Abstract

Eyes with maculopathy usually have poor fixation stability (FS) and develop a new preferred retinal locus (PRL). The exact FS and PRL have never been studied in diabetic macular ischemia (DMI). In this one-year observational study, we recruited 79 patients (145 eyes) with evidence of DMI on optical coherence tomography angiography (OCTA). Microperimetry (MP) was performed at baseline and 52 weeks. Overall, DMI eyes demonstrated relatively stable FS without evolving into eccentric fixation over one year. When comparing the better-seeing eye (BSE) with the worse-seeing eye (WSE) in eyes with bilateral DMI, the latter presented with a larger bivariate contour ellipse area (BCEA) initially but gradually aligned with the one in the BSE at the end of the study. Conversely, the foveolar retinal sensitivity (RS) worsened significantly alongside the extension of disorganization of the retinal inner layers (DRIL) in the WSE at one year despite the best-corrected visual acuity (BCVA) being maintained. This suggests that foveolar RS might reflect the start of DMI deterioration more sensitively than BCVA.

## 1. Introduction

Diabetic retinopathy (DR) is a common microvascular complication in diabetes [1,2]. Depending on the disease severity, 45% to 77% of DR patients may develop diabetic macular ischemia (DMI), a complication that leads to irreversible visual loss [3]. DMI is characterized structurally by disruption of the parafoveal capillary margin, resulting in irregularity and enlargement of the foveal avascular zone (FAZ) [4,5,6,7]. More often, patients with maculopathy develop pseudofovea, also known as preferred retinal locus (PRL), to perform visual tasks [8]. Objectively, PRL can be presented as the absolute location on the retina, and its ability to maintain the same location is known as fixation stability (FS) [8].

Microperimetry (MP) is one of the proposed non-invasive and rapid visual function tests that can help locate the PRL in DMI [8,9]. Similar to a visual field test, MP measures retinal sensitivity (RS) at different locations of the macula, enabling topographic correlation of structure and function in DMI [10,11,12,13,14]. Such structure–function correlations are of invaluable importance for clinical trial designs. Currently, there are five types of MP testing devices commercially available for scientific studies: MP1, MP3, Optos, Compass, and the Macular Integrity Assessment (MAIA). Among these, MAIA is a valid tool approved by the Food and Drug Administration (FDA) for providing fixation indexes, including PRL, P1, P2, and bivariate contour ellipse area (BCEA) [15].

The PRL is defined as the average of locations where the examined eyes lay their focus during testing [8]. In MAIA, PRLi means the initial 250 fixation points acquired during the registration phase, while PRLf represents the ones obtained during the stimuli projection phase. Most of the PRLs in healthy eyes are located at 15.5 degrees horizontally and −1.3 degrees vertically from the middle of the optic disc [8]. Conversely, in eyes with a central lesion engulfing the fovea, the PRL is usually found at the border of the scotoma [8]. Interestingly, the PRL is usually taken by the better-seeing eye (BSE) to facilitate the ocular motor adaptation [8]. A study has shown that visual acuity (VA) is positively correlated with fixation stability only in the better-seeing eye during either monocular or binocular viewing [16,17]. With disease progression, the PRL in the better-seeing eye moves farther in eccentricity but along the same meridian [18].

Fixation stability is defined as the ability of an individual to fixate at a point during the test [12,15,19]. It can be measured using two methods of calculation. The first method, suggested by Fujii et al., counts the percentage of fixation points within 2° and 4° diameter circles, also known as P1 and P2, respectively [20]. If P1 is more than 75%, it is considered a stable fixation. If P1 is less than 75% and P2 is more than 75%, it is relatively unstable. Finally, if both P1 and P2 are less than 75%, it means unstable fixation. While such classification is clinically relevant, it has received criticisms from researchers for being arbitrarily formed [21]. More importantly, disease progression might be missed because of its crude classification [8].

The other calculation method is BCEA, proposed by Crossland et al. [21]. The BCEA is an elliptical area encompassing a given proportion of fixation points (95% or 63%) programmed into MP devices. It is found that the smaller the BCEA, the higher the fixation stability. As BCEA reflects a better score in reading tests than P1 and P2, it is currently considered the gold standard for measuring fixation stability [21]. Morales et al. suggested BCEA@95% of 2.4 ± 2.0 deg^2^ as the normal reference range for fixation stability. The study also found the normal range for BCEA@63% is 0.8 ± 0.7 deg^2^, P1 is 95 ± 5.3%, and P2 is 99 ± 1.4% [13]. Though some studies have confirmed that the BCEA in normal subjects is significantly smaller than that of individuals with other maculopathies, the exact fixation stability range for DMI has never been studied [22].

Currently, there are no approved treatments for DMI [23]. Recently, several new drugs have been investigated, for example, in the HORNBILL and PARTRIDGE clinical trials (NCT04919499 and NCT04424290) [3,24,25]. However, a reliable and clinically meaningful trial endpoint is still lacking. Our previous study result showed the foveal avascular zone (FAZ) area on optical coherence tomography angiography (OCTA) deteriorated constantly over 12 months in DMI [26]. Unfortunately, changes in VA in DMI do not correlate well with the foveal changes identified on OCTA [27]. Upon reviewing the literature, we noticed one study on Best Vitelliform Macular Dystrophy suggested a linear association between fixation stability and best-corrected visual acuity (BCVA) [28]. In addition, the other study on age-related macular degeneration found that fixation stability was significantly associated with reading speed [29]. Therefore, we would like to test the feasibility of these surrogate biomarkers on MP to monitor the progress in DMI.

Overall, this study aimed to (1) report the fixation stability characteristics in DMI eyes with mild to moderate visual impairment; (2) compare the fixation stability indexes between the better-seeing eye (BSE) and worse-seeing eye (WSE) at baseline; and (3) compare the changes in MP, OCT, and OCTA between the better-seeing eye and worse-seeing eye at one year.

## 2. Materials and Methods

### 2.1. Study Design

This prospective one-year cohort study was conducted at Moorfields Eye Hospital between December 2019 and March 2023. The study complies with the tenets of the Declaration of Helsinki and was approved by the United Kingdom (UK) National Research Ethics Committee Service (19/NI/0030). Written consent was acquired from all patients.

### 2.2. Participants

Patients with DR were included if they had at least one eye with the following: (1) evidence of DMI on OCTA, i.e., manually corrected FAZ of at least 0.5 mm^2^ or parafoveal capillary dropout present in at least one quadrant if FAZ falls below 0.5 mm^2^, and (2) best corrected visual acuity (BCVA) of no less than 54 Early Treatment Diabetic Retinopathy Study (ETDRS) letters (Snellen equivalent 20/80). Both eyes were included if they satisfied the eligibility criteria. Patients were scheduled for one baseline and one exit visit at 52 weeks.

The key exclusion criteria were eyes with any conditions that might affect BCVA, such as visually disabling cataracts, as perceived by the investigator; a signal < 20 dB or evidence of center-involved, parafoveal, perifoveal diabetic macular edema (DME) on OCT; having received intravitreal injection within the past six months; fixation losses of 30% or more on MP (as this indicates an unreliable test result based on the MAIA user manual [30]); a quality score < 5 on OCT-A (as this implies significant artifacts with possibly wrong readings [31]); and patients who did not complete the study visits.

### 2.3. Visual Acuity Measurement

Using a high-contrast ETDRS chart (Precision Vision, Bloomington, IL, USA) at 4 m, a masked optometrist examined the patients’ BCVA after formal refraction. The low-luminance visual acuity (LLVA) was assessed by placing a neutral density filter before the ETDRS chart, thereby reducing luminance by 2 log units while maintaining the lighting conditions [32,33]. The BCVA and LLVA for each eye were recorded as the total ETDRS letters the patient could read.

### 2.4. Microperimetry

**Figure 1:**
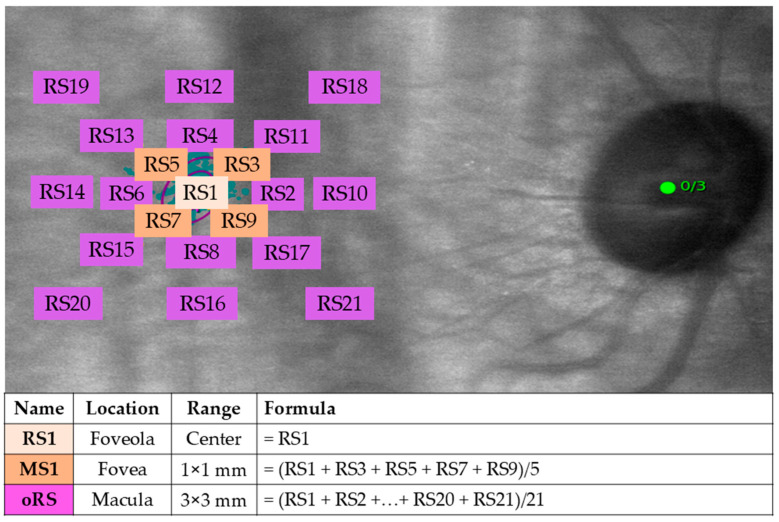
A customized 21-point grid to measure retinal sensitivity at different areas of the retina (right eye as an example). Abbreviations: MS1 = mean sensitivity region 1; oRS = overall retinal sensitivity; RS1 = retinal sensitivity point 1.

Microperimetry was conducted using the Macular Integrity Assessment (MAIA; CenterVue, Padova, Italy). A grid of 21 pointwise retinal sensitivity (RS) in an area of 3 × 3 mm macula (9° around the foveola) was applied (Figure 1). The maximal luminance level was set at 36 dB, and a standard 4-2 strategy was employed as the projection strategy. A Goldmann III stimulus, on a dim white background (1.27 candela/m^2^ or four apostilbs [asb]), with a duration of 200 milliseconds (ms), was used. The speed of fixation tracking was fixed at 25 Hz (every 40 ms). Ten minutes of dark adaptation and a training session were given before formal testing in a mesopic environment. In brief, MAIA has this default setting by giving eight training stimuli to the first-timer [30]. If the patient responds to two of them, the patient understands how to perform the test, and the true test will start from then. The non-study eye was tested first, or the right eye if both eyes were eligible. After averaging all the fixation points recorded during the examination, the machine automatically determined an initial PRL (PRLi) and a final PRL (PRLf). An eccentric fixation was defined as a distance between the PRLf and the estimated foveola location (EFL) of 2° or more [28].

### 2.5. Optical Coherence Tomography

A Spectralis HRA-OCT (Heidelberg Engineering, Heidelberg, Germany) with the settings of 20 × 20 cube volume, 49 raster lines, 1064 pixel resolution, and a speed of 40,000 scans/second was utilized to examine the patient’s macula. The central subfield thickness (CST) was the auto-reading from the centered 1 mm diameter ETDRS grid. We also evaluated the disorganization of the retinal inner layers (DRIL) across the central 1 × 0.72 mm area. DRIL was defined as >3500 mm in the sum of ambiguous boundaries between the ganglion cell layer, inner plexiform layer, inner nuclear layer, and outer plexiform layer.

### 2.6. Optical Coherence Tomography Angiography

A commercial spectral-domain OCT-A (Avanti RTVUE-XR; Optovue, Fremont, CA, USA, version 2018.1.1.60) was used to obtain microvascular parameters from the 3 × 3 mm macular region. The automatic readout of superficial vessel density (SVD) and deep vessel density (DVD) was recorded after correction of the segmentation and decentration errors. The foveal avascular zone (FAZ) was also obtained after manual correction of the delineation.

### 2.7. Statistical Analysis

Patients’ demographics and ocular characteristics were presented as numbers with percentages or means with standard deviations where applicable. A paired *t*-test was used when comparing a continuous variable between eyes in the same patient at different time points. Significance is defined as a *p*-value less than 0.05. All the statistical analyses were carried out using Microsoft Excel (Microsoft 365). Figures were created using the ggplot package in R.

## 3. Results

### 3.1. Demographic and Ocular Characteristics

**Figure 2:**
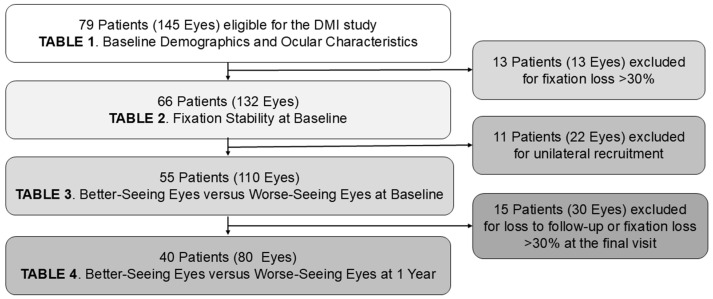
A flowchart showing how participants were included and excluded.

Seventy-nine participants (145 eyes) attended the baseline visit (Figure 2). The average age was 57.8 ± 11.7 years, and more than half of them (62%) were male (Table 1). There were more with type 2 diabetes mellitus (DM) compared to type 1 (58% vs. 42%, respectively), and the average diabetes duration was 28.3 ± 13.6 years. Among these, stable-treated proliferative DR (PDR) accounted for the majority (87%) of the cohort, with ≤5% having active PDR and non-PDR (NPDR).

### 3.2. Baseline Fixation Stability

As per protocol, 13 patients (13 eyes) were excluded because of fixation loss > 30%, leaving 66 patients (132 eyes) entering the baseline analysis (Table 2). These eyes had an average BCVA of 78 ± 9 and LLVA of 69 ± 10 ETDRS letters. The mean overall retinal sensitivity (oRS) across 21 loci was 23.7 ± 4.1 dB. In general, 99% of them exhibited fairly stable FS. When looking at different FS parameters, approximately 50% of them presented with abnormal BCEA@63%, BCEA@95%, and P1, whereas 75% showed abnormal P2. Regarding PRL location, around one-fourth of them were distributed at superotemporal, inferotemporal, and inferonasal parafovea, with an average eccentric distance of 0.18 ± 0.14°.

### 3.3. Fixation Stability Between Eyes at Baseline

At baseline, 55 participants were recruited bilaterally (Table 3). Using fellow eyes as a comparison, the better-seeing eye presented with better BCVA and LLVA than the worse-seeing eye (*p* < 0.001). However, no significant RS difference was found between eyes in the macula, fovea, and foveola. The better-seeing eye had a shorter distance between PRLi and PRLf (0.25 ± 0.15° vs. 0.32 ± 0.22°, *p* = 0.046) than the worse-seeing eye. When examining further, the worse-seeing eye displayed a remarkably larger BCEA@63% (0.99 ± 0.90 deg^2^ vs. 0.76 ± 0.55 deg^2^, *p* = 0.03) and BCEA@95% (2.97 ± 2.70 deg^2^ vs. 2.29 ± 1.68 deg^2^, *p* = 0.03). In contrast, the worse-seeing eye possessed a significantly smaller proportion of P2 (98.7 ± 2.3% vs. 99.5 ± 1.2%, *p* = 0.001). Although there was a trend suggesting more DRIL in the worse-seeing eye, it did not reach statistical significance, nor did the rest of the macular parameters, such as CST, FAZ, SVD, and DVD.

### 3.4. Fixation Stability Between Eyes at One Year

**Figure 3:**
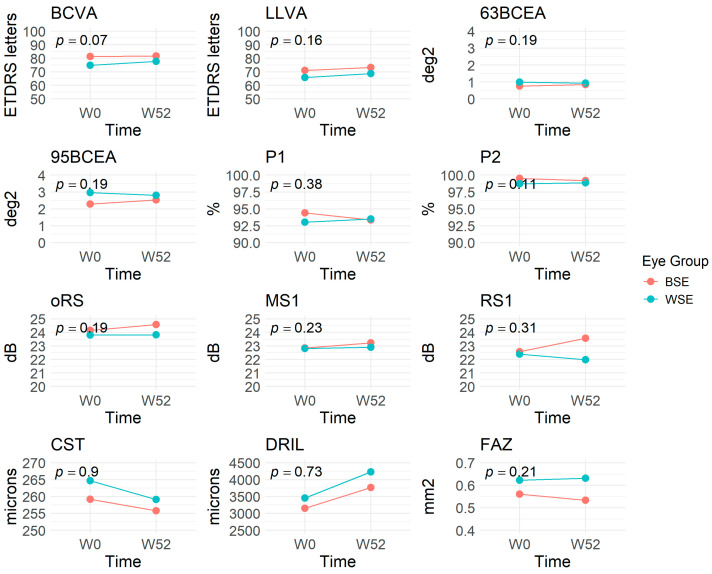
A combined line chart demonstrating the trend of different functional and anatomical parameters in eyes with diabetic macular ischemia over one year. **Key note:** Most parameters presented similar changes in BSE and WSE. The only exception was RS1, where the BSE gained retinal sensitivity, but the WSE lost retinal sensitivity, resulting in a significant difference between eyes at 12 months. Abbreviations: BCEA = bivariate contour ellipse area; BCVA = best-corrected visual acuity; BSE = better-seeing eye; CST = central subfield thickness; DRIL = disorganization of retinal inner layers; FAZ = foveal avascular zone; LLVA = low-luminance visual acuity; MS1 = mean sensitivity region 1; oRS = overall retinal sensitivity; P1 = percentage of fixation points within the 2° diameter circle; P2 = percentage of fixation points within the 4° diameter circle; RS1 = retinal sensitivity point 1; WSE = worse-seeing eye. The *p*-values were derived from paired *t*-tests comparing changes over time in the parameters between BSE and WSE eyes.

Forty patients with 80 eyes completed the one year exit visit (Table 4). When comparing both eyes, the better-seeing eye at baseline still maintained a significantly better BCVA and LLVA (*p* < 0.001) without remarkable changes. There was a non-significant decrease in the worse-seeing eye’s BCEA and an increase in the better-seeing eye’s BCEA, either BCEA@63% or BCEA@95%, balancing the differences in BCEA between both eyes at baseline (*p* > 0.05) (Figure 3). Meanwhile, the PRL still remained less than 2° from the EFL without developing eccentric fixation. Looking closely at the RS changes, the worse-seeing eye presented with gradual loss of RS in oRS, MS1, and RS1, while the RS in the better-seeing eye improved, resulting in a noticeable difference in the final RS1 between eyes at one year (22.0 ± 4.3 dB in worse-seeing eyes vs. 23.6 ± 3.9 dB in better-seeing eyes, *p* = 0.04). At one year, the DRIL in the worse-seeing eye progressed to 4226 ± 1945 µm, which was statistically and significantly more than the better-seeing eye (*p* = 0.049). In addition, the worse-seeing eye’s FAZ showed a trend to enlarge with a slight reduction in the better-seeing eye, but the final FAZ at 52 weeks did not show a significant difference (*p* = 0.15). The CST, SVD, and DVD did not change much between eyes during the one year observation period.

## 4. Discussion

In this prospective study, we examined visual function against FS, PRL, RS, and anatomical biomarkers between both eyes in DMI patients over 1 year. We found several significant findings. Firstly, DMI eyes did not present with eccentric fixation despite an enlarged FAZ on OCTA, and their fixation stability was predominantly stable, although approximately 50% of these eyes had abnormal BCEA@63% and BCEA@95%. Moreover, the worse-seeing eye showed a more extensive BCEA@63% and BCEA@95% compared to the better-seeing eye at the baseline. After one year of observation, the difference in BCEA between both eyes was minimized. However, foveolar RS and DRIL worsened in the worse-seeing eye, resulting in a significant difference between both eyes (Figure 3).

When examining the fixation stability in DMI using the grading system proposed by Fujii et al., we found that nearly all eyes had a stable fixation stability and remained the same throughout the year, implying that fixation stability is not an ideal clinical trial endpoint for DMI. This is extremely different from the scenarios in advanced ARMD, where 68% of the eyes present with unstable fixation stability [29]. Moreover, eyes with DMI had their PRL very close to the EFL (0.18 ± 0.14°), far less than those with advanced ARMD (5.15 ± 3.31°) [29], and none of them fulfilled the criterion of eccentric fixation (≥2°) [28]. Together, these findings corroborate the proposal that fixation stability is positively correlated with the eccentricity distance between PRL and EFL [16,22,28,29,34]. They also indicate that DMI eyes have relatively stable fixation stability, and eccentric fixation is rarely seen.

We also looked at the newer fixation stability grading system advocated by Crossland et al. We saw approximately 50% of the DMI eyes had abnormal BCEA@63% and BCEA@95% if we applied the normal threshold suggested by Morales et al. We would expect these eyes to have a slower reading speed [21,29,35,36], though this test was not listed in our protocol. However, we did observe these eyes, despite having preserved BCVA, as presenting with difficulties staying still when obtaining OCTA images clinically, especially the 6×6 mm macular scan, which requires a longer image-acquisition time.

When we compared the better-seeing eyes with the worse-seeing eyes in our DMI cohort, the worse-seeing eyes showed significantly larger BCEA@63% and BCEA@95%. Similarly, Bianco et al. reported that BCVA could be worsened by 0.01 LogMAR (equivalent to 0.5 ETDRS letters) with every 1 deg^2^ increase in BCEA@95% [28]. Together, we support the concept that VA is positively correlated with fixation stability. In addition, we also noticed that the better-seeing eye had a shorter distance between PRLi and PRLf than the worse-seeing eye. This finding indicates quicker adaptation to the light stimulation in the better-seeing eye, and perhaps it could be used to explain why the better-seeing eye drives the binocular control [8].

With time, we found that eccentricity distance, either in the better-seeing eye or the worse-seeing eye in DMI, did not change significantly. This finding is in contrast to a previous report on maculopathy, where the PRL in the better-seeing eye moved farther in eccentricity but along the same meridian [8,18]. Moreover, we found a trend of “uniting” BCEA between eyes, resulting in no significant difference in BCEA at one year. It seems that the better-seeing eye was compensating or helping the worse-seeing eye to reach a balance between the eyes. However, the foveolar RS (RS1) in the worse-seeing eye still worsened at one year despite maintaining a similar BCVA. We know from our previous report that the correlation between BCVA and RS is modest [37]. Taking this finding into the present study, the RS1 may be a better biomarker than BCVA to reflect the early downfall of DMI, especially in the worse-seeing eye.

We would like to highlight the strengths of this study. To the best of our understanding, this is the first study to look at the fixation stability and PRL changes in eyes with DMI over a year, enabling us to provide clinical trial designers with useful biomarker references. We also applied clear and objective definitions of DMI, so there was no ambiguity in patient selection. Furthermore, all participants received MP training in every session before the formal examination, thereby reducing measurement errors. Finally, the MAIA machine we employed had inbuilt software to automatically remove the PRL outliers [8], improving the final data reliability.

We also acknowledge some limitations. First, the patients we included were not treatment-naïve; however, we excluded those with recent injections to ensure there were no confounders in the cohort. Second, we recruited patients with only mild to moderate visual impairment to yield more valid MP results. Nevertheless, eyes with poor vision would be worth exploring in the future. Third, we tested the PRL under monocular viewing conditions due to technical constrictions; however, it would be ideal to compare the results with both eyes open, as the better-seeing eye usually drives the PRL, theoretically [8].

## 5. Conclusions

This one-year observational study demonstrates that DMI eyes have relatively stable fixation stability and seldom develop eccentric fixation. Although the worse-seeing eye presented with larger BCEA initially, both eyes ended with similar BCEA at the end. The observed decrease in foveolar RS, particularly at point RS1, suggests it could serve as an early indicator of worsening visual function in DMI. These findings highlight the potential of RS1 as a key biomarker, offering valuable insights that could guide the development of more targeted treatment strategies and inform clinical trial designs for DMI patients.

## Figures and Tables

**Table 1:** Baseline demographics and ocular characteristics.

Patients	All Participants (*n* = 79)
Age, mean (SD), years	57.8 (11.7)
Sex	
Male, *n* (%)	49 (62%)
Female, *n* (%)	30 (38%)
Diabetes	
Type 1, *n* (%)	33 (42%)
Type 2, *n* (%)	46 (58%)
Diabetes duration, mean (SD), years	28.3 (13.6)
Bilateral recruitment, *n* (%)	66 (84%)
**Ocular characteristics**	**All eligible eyes (*n* = 145)**
Diabetic retinopathy severity	
Active PDR, *n* (%)	4 (3%)
Stable-treated PDR, *n* (%)	126 (87%)
Severe NPDR, *n* (%)	7 (5%)
Moderate NPDR, *n* (%)	6 (4%)
Mild NPDR, *n* (%)	2 (1%)
Examinations	
Intraocular pressure, mean (SD), mmHg	15 (4)
Spherical equivalent, mean (SD), diopter	−0.32 (2.09)
Past ocular history	
Cataract surgery, *n* (%)	53 (37%)
Pars plana vitrectomy, *n* (%)	26 (18%)
Diabetic macular edema, *n* (%)	24 (17%)
Macular Laser, *n* (%)	31 (21%)
Microperimetry	
Fixation loss > 30%, *n* (%)	13 (9%)

Abbreviations: NPDR = non-proliferative diabetic retinopathy, PDR = proliferative diabetic retinopathy, SD = standard deviation.

**Table 2:** Fixation stability at baseline.

Microperimetry	Eyes (*n* = 132)
Vision	
BCVA, mean (SD), letters	78 (9)
LLVA, mean (SD), letters	69 (10)
Fixation loss, median, (Q1, Q3), %	0 (0.0)
PRL	
PRL distance (i, f), mean (SD), °	0.29 (0.19)
Eccentric distance (PRLf, EFL), mean (SD), °	0.18 (0.14)
Superior, *n* (%)	3 (2%)
Superotemporal, *n* (%)	30 (23%)
Temporal, *n* (%)	3 (2%)
Inferotemporal, *n* (%)	36 (27%)
Inferior, *n* (%)	1 (1%)
Inferonasal, *n* (%)	37 (28%)
Nasal, *n* (%)	2 (2%)
Superonasal, *n* (%)	20 (15%)
BCEA@63%, mean (SD), deg^2^	0.97 (0.81)
Abnormal (>0.8 deg^2^), *n* (%)	69 (52%)
BCEA@95%, mean (SD), deg^2^	2.91 (2.44)
Abnormal (>2.4 deg^2^), *n* (%)	64 (48%)
P1, mean (SD), %	92.9 (6.5)
Abnormal (<95%), *n* (%)	66 (50%)
P2, mean (SD), %	98.8 (2.1%)
Abnormal (<99%), *n* (%)	99 (75%)
Fixation stability	
Stable, *n* (%)	131 (99%)
Relative unstable, *n* (%)	1 (1%)
Unstable, *n* (%)	0 (0%)
Retinal sensitivity	
oRS (macula), mean (SD), dB	23.7 (4.1)
MS1 (fovea), mean (SD), dB	22.5 (4.8)
RS1 (foveola), mean (SD), dB	22.2 (4.5)

Abbreviations: BCEA = bivariate contour ellipse area; BCVA = best-corrected visual acuity; f = final; EFL = estimated foveola location; i = initial; LLVA = low-luminance visual acuity; MS1 = mean sensitivity region 1; oRS = overall retinal sensitivity; P1 = percentage of fixation points within the 2° diameter circle; P2 = percentage of fixation points within the 4° diameter circle; PRL = preferred retinal loci; PRLf = final preferred retinal loci; RS1 = retinal sensitivity point 1.

**Table 3:** Better-seeing eyes versus worse-seeing eyes at baseline.

Bilateral Recruitment (*n* = 55)	BSE	WSE	*p*-Value
Vision	Mean (SD)	Mean (SD)	
BCVA (letters)	81 (7)	75 (10)	<0.001
LLVA (letters)	71 (9)	66 (11)	<0.001
Microperimetry			
PRL distance (i, f) (°)	0.25 (0.15)	0.32 (0.22)	0.046
Eccentric distance (PRLf, EFL) (°)	0.17 (0.15)	0.19 (0.15)	0.53
BCEA@63% (deg^2^)	0.76 (0.55)	0.99 (0.90)	0.03
BCEA@95% (deg^2^)	2.29 (1.68)	2.97 (2.70)	0.03
P1 (%)	94.4 (5.4)	93.0 (6.6)	0.07
P2 (%)	99.5 (1.2)	98.7 (2.3)	0.001
oRS (macula) (dB)	24.1 (4.2)	23.8 (3.9)	0.45
MS1 (fovea) (dB)	22.8 (5.0)	22.8 (4.3)	0.96
RS1 (foveola) (dB)	22.6 (4.0)	22.4 (4.2)	0.77
OCT			
CST (µm)	259 (29)	265 (43)	0.22
DRIL (µm)	3153 (1800)	3458 (1919)	0.06
OCTA ^a^			
FAZ (mm^2^)	0.56 (0.39)	0.62 (0.37)	0.29
SVD (%)	37.2 (4.8)	36.8 (5.1)	0.49
DVD (%)	43.0 (4.8)	42.7 (4.4)	0.66

Key notes: The worse-seeing eyes presented with a larger bivariate contour ellipse area and a smaller P2, indicating less fixation stability than the better-seeing eyes. Abbreviations: BCEA = bivariate contour ellipse area; BCVA = best-corrected visual acuity; BSE = better-seeing eye; CST = central subfield thickness; DRIL = disorganization of retinal inner layers; DVD = deep vessel density; f = final; FAZ = foveal avascular zone; EFL = estimated foveola location; i = initial; LLVA = low-luminance visual acuity; MS1 = mean sensitivity region 1; oRS = overall retinal sensitivity; P1 = percentage of fixation points within the 2° diameter circle; P2 = percentage of fixation points within the 4° diameter circle; PRL = preferred retinal loci; RS1 = retinal sensitivity point 1; SVD = superficial vessel density; WSE = worse-seeing eye. ^a^ N = 50 after excluding scans with poor quality.

**Table 4:** Better-seeing eyes versus worse-seeing eyes at one year.

At One Year (*n* = 40)	BSE at Baseline	WSE at Baseline	*p*-Value
**Vision**	Mean (SD)	Mean (SD)	
BCVA (letters)	82 (8)	78 (9)	<0.001
BCVA changes (letters)	0 (3)	2 (5)	0.07
LLVA (letters)	73 (8)	69 (9)	<0.001
LLVA changes (letters)	1 (5)	2 (7)	0.16
**Microperimetry**			
PRL distance (i, f) (°)	0.28 (0.17)	0.31 (0.17)	0.22
PRL distance (i, f) changes (°)	0.01 (0.20)	0.01 (0.20)	0.95
Eccentric distance (PRLf, EFL) (°)	0.20 (0.25)	0.22 (0.20)	0.69
Eccentric distance (PRLf, EFL) changes (°)	0.01 (0.27)	0.04 (0.17)	0.55
BCEA@63% (deg^2^)	0.85 (0.67)	0.93 (0.76)	0.45
BCEA@63% changes (deg^2^)	0.06 (0.63)	−0.08 (0.72)	0.19
BCEA@95% (deg^2^)	2.53 (2.01)	2.80 (2.29)	0.43
BCEA@95% changes (deg^2^)	0.16 (1.89)	−0.22 (2.14)	0.19
P1 (%)	93.4 (6.4)	93.5 (6.5)	0.89
P1 changes (%)	−0.6 (5.5)	0.2 (5.7)	0.38
P2 (%)	99.2 (1.8)	98.9 (1.9)	0.36
P2 changes (%)	−0.2 (2.1)	0.4 (2.2)	0.11
oRS (dB)	24.6 (4.1)	23.8 (4.1)	0.19
oRS changes (dB)	0.4 (2.8)	−0.1 (2.2)	0.19
MS1 (dB)	23.2 (5.5)	22.9 (4.5)	0.63
MS1 changes (dB)	0.5 (3.2)	−0.2 (2.7)	0.23
RS1 (dB)	23.6 (3.9)	22.0 (4.3)	0.04
RS1 changes (dB)	0.7 (5.0)	−0.4 (5.1)	0.31
**OCT**			
CST (µm)	255.8 (29.1)	259.2 (40.0)	0.46
CST changes (µm)	−3.4 (11.7)	−2.9 (22.0)	0.90
DRIL (µm)	3765 (1889)	4226 (1945)	0.049
DRIL (µm)	530 (1083)	595 (976)	0.73
**OCTA** ^a^			
FAZ (mm^2^)	0.533 (0.292)	0.631 (0.390)	0.15
FAZ changes (mm^2^)	0.013 (0.021)	0.026 (0.053)	0.21
SVD (%)	37.6 (3.7)	37.1 (5.4)	0.59
SVD changes (%)	0.0 (3.9)	−0.2 (3.5)	0.75
DVD (%)	43.1 (4.1)	43.0 (4.9)	0.91
DVD changes (%)	−0.5 (4.2)	−0.3 (3.2)	0.83

Key notes: The better-seeing eye continued to gain retinal sensitivity while the worse-seeing eye kept losing retinal sensitivity, resulting in a significant difference in the final RS1 between eyes at 12 months (*p* = 0.04). Abbreviations: BCEA = bivariate contour ellipse area; BCVA = best-corrected visual acuity; BSE = better-seeing eye; CST = central subfield thickness; DRIL = disorganization of retinal inner layers; DVD = deep vessel density; f = final; FAZ = foveal avascular zone; EFL = estimated foveola location; i = initial; LLVA = low-luminance visual acuity; MS1 = mean sensitivity region 1; oRS = overall retinal sensitivity; P1 = percentage of fixation points within the 2° diameter circle; P2 = percentage of fixation points within the 4° diameter circle; PRL = preferred retinal loci; RS1 = retinal sensitivity point 1; SVD = superficial vessel density; WSE = worse-seeing eye. ^a^ N = 34 after excluding scans with poor quality.

## Data Availability

Sivaprasad has full access to all the data in the study and takes responsibility for both the integrity of the data and the accuracy of the data analysis. The data will be made available upon request (sobha.sivaprasad@nhs.net).
